# Silencing of the DNA damage repair regulator *PPP1R15A* sensitizes acute myeloid leukemia cells to chemotherapy

**DOI:** 10.1007/s00277-024-05785-x

**Published:** 2024-06-06

**Authors:** Anthi Bouchla, Christina D. Sotiropoulou, Christopher Esteb, Theodoros Loupis, Sotirios G. Papageorgiou, Georgia G. Deliconstantinos, Maria Pagoni, Eleftheria Hatzimichael, Maria Dellatola, Smaragdi Kalomoiri, Elisavet Apostolidou, Christos K. Kontos, Thomas P. Thomopoulos, Theodoros Karantanos, Vasiliki Pappa

**Affiliations:** 1 Second Department of Internal Medicine and Research Institute, National and Kapodistrian University of Athens, Athens, Greece; 2 Department of Biochemistry and Molecular Biology, Faculty of Biology, National and Kapodistrian University of Athens, Athens, Greece; 3 Hematologic Malignancies Sidney Kimmel Comprehensive Cancer Center, Johns Hopkins University, Baltimore, MD USA; 4 Hematology Research Lab, Clinical, Experimental and Translational Research Center, Biomedical Research Foundation, Academy of Athens, Athens, Greece; 5 Hematology-Lymphomas Department and BMT Unit, Evangelismos General Hospital, Athens, Greece; 6 Department of Hematology, University Hospital of Ioannina, Ioannina, Greece

**Keywords:** DNA damage response, GADD34, PPP1R15A, Acute myeloid leukemia, Resistance mechanisms

## Abstract

Acute Myeloid Leukemia (AML) is a life-threatening disease whose induction treatment consists of combination chemotherapy with Idarubicin and Cytarabine for fit patients. Treatment failures are frequent, urging the need for novel treatments for this disease. The DNA Damage Response Mechanism (DDR) comprises numerous molecules and pathways intended to arrest the cell cycle until DNA damage is repaired or else drive the cell to apoptosis. AML-derived cell lines after treatment with Idarubicin and Cytarabine were used for studying the expression profile of 84 DDR genes, through PCR arrays. Utilizing de novo AML patient and control samples we studied the expression of *PPP1R15A, CDKN1A, GADD45A, GADD45G*, and *EXO1.* Next, we performed *PPP1R15A* silencing in AML cell lines in two separate experiments using siRNA and CRISPR-cas9, respectively. Our findings highlight that DDR regulators demonstrate increased expression in patients with high cytogenetic risk possibly reflecting increased genotoxic stress. Especially, *PPP1R15A* is mainly involved in the recovery of the cells from stress and it was the only DDR gene upregulated in AML patients. The *PPP1R15A* silencing resulted in decreased viability of Idarubicin and Cytarabine-treated cell lines, in contrast to untreated cells. These findings shed light on new strategies to enhance chemotherapy efficacy and demonstrate that *PPP1R15A* is an important DDR regulator in AML and its downregulation might be a safe and effective way to increase sensitivity to chemotherapy in this disease.

**Supplementary Information:**

The online version contains supplementary material available at 10.1007/s00277-024-05785-x.

## Introduction

Acute Myeloid Leukemia (AML) is a life-threatening disease that is currently treated with intensive chemotherapy in eligible patients of young age with no substantial comorbidities. Even though AML is a highly heterogeneous disease, the backbone of induction chemotherapy remains the combination of Idarubicin (or Doxorubicin) and Cytarabine. Both these agents act by causing DNA damage to the leukemic cells, thereby eliminating them [1, 2]. Although effective, a considerable proportion of patients do not respond to induction therapy or lose initial response, resulting in 5-year survival rates of less than 60% in patients up to 60 years of age [3]. Understanding the pathophysiology of the disease and mechanisms of resistance to induction chemotherapy is crucial for developing new targeted therapies that can improve the response rates and overall survival of AML patients.

DNA Damage Response (DDR) represents an attractive pathway for translational research in cancer therapeutics. It refers to an intricate mechanism of responding to DNA damage once it occurs. Comprising numerous molecules and pathways, its main function is to prevent damage expansion by arresting the cell cycle until the damage is repaired, or else driving cells to apoptosis. DDR can act as a double-edged sword, in the sense that impaired DDR may lead to leukemogenesis, while hyperactivated DDR may reverse chemotherapy-induced DNA damage and result in chemoresistance [4]. Although more extensively studied in solid malignancies, DDR has also emerged as an important mechanism in the pathophysiology of hematopoietic malignancies. DDR inhibition in combination with conventional treatment is also being investigated as a means of inducing synthetic lethality in hematological malignancies [4, 5].

Defective DDR has been involved in AML pathogenesis. Hematopoietic stem cells are prone to the accumulation of genetic lesions owing to their long lifespan and repeated exposure to stressful conditions that force them to enter the cell cycle resulting in replication stress [6]. Specific entities related to DDR impairment such as congenital DDR deficiencies [7], inherited and somatic TP53 mutations [8], and multiple polymorphisms in DDR genes [9] have been associated with increased AML incidence.

In this study, we showed that several DDR genes are upregulated in cell lines following treatment with Idarubicin and Cytarabine. One of them, *PPP1R15A*, was upregulated in AML patients compared to controls, and its knockdown by two different methods resulted in increased AML chemosensitivity.

## Materials and methods

### Patient and control samples

Mononuclear cells were isolated from bone marrow samples from 74 patients with de novo AML at diagnosis and 30 lymphoma subjects without evidence of bone marrow involvement - the latter were used as healthy controls. The research was approved by the institutional review board of Attikon University Hospital (ΕΒΔ2421/26-05-2017) and all participants gave written informed consent in agreement with the Declaration of Helsinki. All AML patients had received one to two induction cycles of Cytarabine and Idarubicin and response to treatment was evaluated after the end of induction. Isolation was performed with Lymphoprep (STEM CELL Technologies) by density gradient centrifugation. Isolated cells in RPMI were stored in cryovials at – 80˚C. Mononuclear cells were homogenized using a QIA shredder spin column (Qiagen Ltd., Hilden, Germany).

### Cell culture

Four AML cell lines namely MV-4-11, MOLM-13 (MLL-AF9 fusion, FLT3-ITD mutated), KASUMI-1 (core binding factor AML), and TF-1 (erythroleukemia), were cultured based on ATCC® guidelines. The growth conditions were 5% CO2, 95% air, and a temperature of 37℃.

### MTT assay

Cell lines KASUMI-1 and MV-4-11 were seeded in a 96-well plate at a concentration of 1 × 10^5^ cells/mL, 16 h after seeding they were treated based on bibliographic data with Idarubicin and Cytarabine in order to conduct the gene expression profiling. MOLM-13 and KASUMI-1 cells were seeded in a 96-well plate at a concentration of 1 × 10^5^ cells/mL. Afterward, they were treated with Idarubicin or Cytarabine at a concentration range of 1nM- 10µΜ (with a 10fold gradual increase) and were further incubated for 24, 48, and 72 h. TF-1 cells were seeded in a 96-well plate at a concentration of 1 × 10^5^ cells/mL. Afterward, they were treated with Idarubicin (0.01, 0.05, 0.1, 0.2, and 0.5µΜ) or Cytarabine (0.1, 0.5, 01, 2, 5µΜ) and were further incubated for 24, 48 and 72 h. The colorimetric 3-(4, 5-dimethylthiazol-2-yl)-2, 5-diphenyl-tetrazolium bromide (MTT) assay was used for cell viability determination at each time-point and for each agent concentration. MTT (Sigma, USA) was dissolved in PBS at 5 mg/mL, the MTT solution was added to the 96-well plate at volume 20 µL, and the resulting solution was incubated in 5% CO2 for another 4 h at 37 °C. Formazan crystals were dissolved in 200 µL of SDS-HCL. The plates were then analyzed in a plate reader at 570 nm. All experiments were performed in triplicate.

### Trypan blue assay

Trypan Blue assay was also used to determine the number of viable cells after treatment of both cell lines with the different concentrations of Idarubicin or Cytarabine in 24, 48, and 72 h. MOLM-13 and KASUMI-1 cells were seeded in a 6-well plate at a concentration of 1 × 10^5^ cells/mL, then treated and incubated at three-time points. Next, we mixed 1 part of 0.4% trypan blue (Sigma-Aldrich, Merck) and 1 part cell suspension and the total cells were counted within 3 to 5 minutes with a hemacytometer. All experiments were performed in triplicate.

### The half-maximal inhibitory concentration (IC50) calculation

The IC50 values 24,48 and 72 h after treatment with different concentrations of Idarubicin or Cytarabine in MOLM-13 and KASUMI-1 cell lines were determined by plotting the MTT assay results of % viability vs. time for each drug concentration. Subsequently, cell viability was further assessed using the trypan blue assay to confirm the results.

### Clonogenicity assay

TF-1 cells were counted and re-suspended at a density of 2000 cells/ml in methylcellulose-based media. After 10 to 14 days of incubation at 37 °C in 5% CO2, the recovery of colony-forming units was determined by colony counting under bright-field microscopy. A cell aggregate composed of > 50 cells was defined as a colony.

### siRNA transfection

In 6-well plates, MOLM-13 and KASUMI-1 cells were seeded at the specified concentrations. Following the designated incubation period, treatment with Idarubicin (10 nM) or Cytarabine (100 nM) was administered to MOLM-13, while KASUMI-1 received Idarubicin (1 nM) or Cytarabine (100 nM). Untreated wells were also incorporated. After 24 h, 10 pmol of siRNA was transfected into each respective well. In both treated and untreated cells, wells containing 10 pmol of a scrambled sequence were included, as were wells comprising only the transfection reagents (mock control). Each reaction was conducted in triplicates using the Lipofectamine™ RNAiMAX (Invitrogen™, Thermo Fisher Scientific Inc.). The cells were incubated with the transfection reagents for 24 and 48 h. The principles of the designed sequences are shown in Supplemental Table S1.

### Total RNA isolation and RNA quality control

Total RNA was extracted from patient samples utilizing the RNeasy Plus Mini Kit (Qiagen Ltd., Hilden, Germany). Additionally, total RNA was isolated from transfected cells employing NucleoZOL (MACHEREY-NAGEL GmbH & Co. KG, Düren, Germany). The concentration of total RNA was determined using the BioSpec-nano Micro-volume UV–vis Spectrophotometer (Shimadju, Kyoto, Japan), and their integrity was assessed via electrophoresis in 1.2% agarose gel.

### Reverse transcription and real-time quantitative PCR (qPCR)

200 ng of each RNA extract from patient samples was used as a template for cDNA synthesis. First-strand synthesis was conducted using M-MLV reverse transcriptase (Life Technologies Ltd., Carlsbad, CA, USA) and oligo-dT primer. All reactions were performed according to the manufacturer’s instructions. 100 ng of cDNA of patient samples were diluted in QuantiNova Yellow Template Dilution Buffer were mixed with 10 µl of 2x QuantiNova SYBR Green PCR Master Mix (Qiagen Ltd., Hilden, Germany), 2 µl of 10x QuantiTect primer, and RNase free water. Real-time qPCR assays followed using KAPA™ SYBR® FAST qPCR master mix (2X) (Kapa Biosystems Inc., Woburn, MA, USA). Reactions were performed in a QuantStudio 5 Real-Time PCR System (Applied Biosystems, Thermo Fisher Scientific Inc.), according to the manufacturer’s protocol. Gene expression was normalized against *GAPDH* expression and relative gene expression was calculated by the ΔCt method. We studied gene expression in correlation with disease status, ELN2017 AML cytogenetic risk [10], presence of FMS like Tyrosine kinase 3 mutation Internal Tandem Duplication (FLT3-ITD) and nucleophosmine 1 (NPM1) mutations and patient survival.

To investigate the impact of Idarubicin and Cytarabine at their respective IC50 concentrations on *PPP1R15A* expression in AML cell lines, gene expression analysis was performed using qRT-PCR as described above. Melt curve analysis was also utilized to verify the specificity of each amplification product. For each pairing of cell line and inhibitor, the normalized RQU or fold change values were expressed as log2 fold change. To elucidate the functional significance of *PPP1R15A* in AML cell viability and chemotherapy response, experiments involving *PPP1R15A* silencing using siRNA in MOLM-13 and KASUMI-1 cell lines were conducted. Initially, the efficiency of *PPP1R15A* silencing by qPCR was assessed by comparing the expression levels to cells transfected with a scramble sequence. Furthermore, cell viability comparisons between *PPP1R15A*-silenced and mock control cells were made after treatment with Cytarabine and Idarubicin at IC50 concentrations for 24 and 48 h. For this purpose, *GADPH*, *HRPT1*, and *B2M* were used as reference genes.

Gene expression profiling through PCR arrays analysis (Qiagen RT² Profiler PCR Array (96-Well Format and 384-Well [4 × 96] Format) Human DNA Damage Signaling Pathway) was performed in triplicate after RNA extraction from untreated, chemotherapy-treated, and live cells following chemotherapy exposure. Human DNA damage signaling pathway-related gene expression was evaluated and analyzed through the RT2 Profiler PCR Array data analysis tool (Rotor-Gene_2_3_5_1) and Fold Change values per condition were calculated using the respective untreated sample as control. All the primer sequences that were used from the above experiments are shown in Supplemental Tables S2 and S3.

### CRISPR-Cas9 mediated PPP1R15A deletion

PLKO.1-puro lentiviral vectors expressing CRISPR-associated endonuclease 9 (Cas9) and two different single guide RNAs (sgRNAs) targeting *PPP1R15A* (sgRNA1: AGGTCCTGGGAGTATCGTTC, sgRNA2: GGACAACACTCCCGGTGTGA) or scramble RNA (GTGTAGTTCGACCATTCGTG) as control purchased from VectorBuilder were transfected into HEK-293FT cells [11]. TF-1 cells were transduced with lentiviruses and selected with puromycin (2 µg/ml). Subsequently, single-cell cloning was done, and clones were screened by performing qPCR to select *PPP1R15A* deleted clones.

### Flow cytometry

KASUMI-1 and MV-4-11 cell sorting was performed using a flow-cytometry-based approach with Annexin V staining. In brief, cultured cells after treatment with either Idarubicin or Cytarabine, washed with ice-cold phosphate-buffered saline (PBS) and resuspended in binding buffer (10 mM HEPES, 140 mM NaCl, 2.5 mM CaCl2, pH 7.4). Subsequently, cells were stained with Annexin V-FITC (fluorescein isothiocyanate) according to the manufacturer’s instructions (BD Biosciences Annexin V-FITC Apoptosis Detection Kit). Sorting gates were established based on the Annexin V-FITC signal intensity and FSC/SSC characteristics to isolate the viable cell population. The entire sorting process was conducted at 4 °C. TF-1 cells were fixed in 4% formaldehyde for 15 min and permeabilized in 100% methanol for 30 min. Subsequently, cells were labeled with the phycoerythrin (PE) conjugated *PPP1R15A* antibody purchased from Santa Cruz (sc-373,815). Gating was based on clearly distinguishable populations, or in the absence of such, the negative antibody control. Median fluorescence intensity (MFI) was determined for each marker using FlowJo analysis software version 10.0.8 (FlowJo, Ashland, CO, USA).

### Western blotting

Cell pellets were homogenized on ice in lysis buffer Nonident P-40 (NP-40) (Sigma-Aldrich, St. Louis, MI, USA), that containing protease inhibitors (Sigma-Aldrich, St. Louis, MO, USA) and centrifuged for 10 min (4 °C) at 20,000× g. Samples were subjected to SDS-PAGE and immunoblotting analysis after the protein content was calibrated for each sample using the Bradford assay (Bio-Rad, Hercules, CA, USA). Using sodium dodecyl sulfate-polyacrylamide gel electrophoresis (SDS-PAGE), 10 micrograms of protein were separated and then transferred to a nitrocellulose membrane (MACHEREY-NAGEL GmbH & Co. KG, Düren, Germany). Following blocking with 5% bovine serum albumin (BSA) (Sigma-Aldrich, St. Louis, MO, USA), the membranes were incubated with primary antibody, GADD34 (E3S6N) Rabbit mAb and β-Actin (13E5) Rabbit mAb (Cell Signaling Technology, Danvers, Massachusetts, USA) overnight at 4 °C, followed by the respective secondary antibodies. Immunoblots were developed using Clarity Western ECL Blotting Substrate (Bio-Rad, Hercules CA, USA). Blot quantification was performed using ImageJ 1.53k (NIH).

### Statistical analysis

To visualize the relative response profile of each cell treatment in the KASUMI-1 and MV4-11 cells, Principal Component analysis (PCA) was performed using the gene expression (2^−ΔCt^) of all 84 DDR genes from the Qiagen’s RT2 Profiler PCR Array. For differential gene expression in patient and control samples, statistical analysis was performed using the Kruskal-Wallis test and Dunn’s test for pairwise multiple comparisons, with a significance level of *p* < 0.05. Correlation analysis was performed using Spearman’s Rho and survival analysis was performed using the Kaplan-Meier method and the log-rank test. Statistical analysis was performed using STATA 17 software.

## Results

### Gene expression profiling

PCA of the 84 genes involved in DNA Damage Response (DDR), revealed distinct patterns of DDR gene expression in MV4-11 and KASUMI-1 cell lines before and after treatment with Idarubicin and Cytarabine. DDR gene expression following treatment was measured in both the entire population of treated cells and the live cells, as seen in Supplemental Figure S1.

**Fig. 1:**
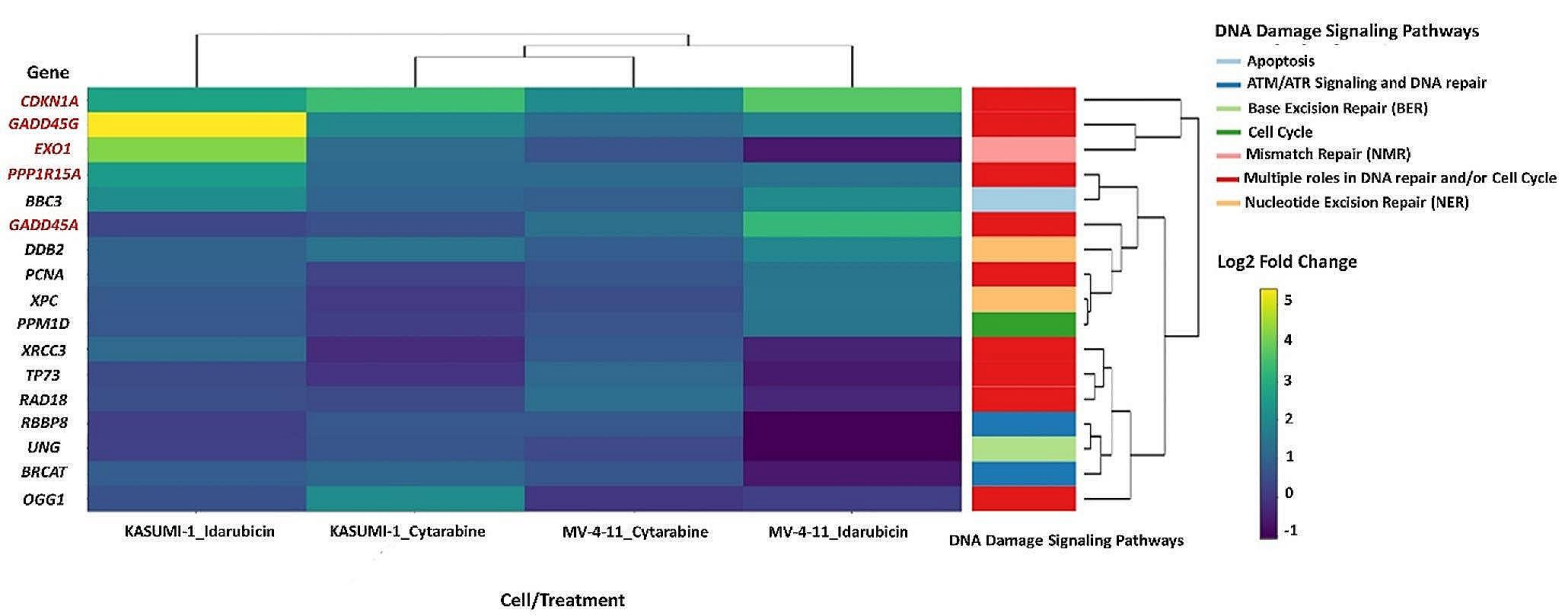
Heatmap of differentially expressed genes with at least one statistically significant fold change (p-value < 0.05) either in KASUMI-1 or MV4-11 live cells treated with Idarubicin or Cytarabine. Marked in bold are the selected genes whose expression was subsequently studied in bone marrow samples of AML patients

The following genes were over two-fold up-regulated in live cells from leukemic cell lines after treatment with both agents: *PPP1R15A*, *CDKN1A*, and *GADD45G* genes were up-regulated in both live cell lines, *GADD45A* in live MV4-11 cells and *EXO1* in live KASUMI-1 cells (Fig. 1).

These genes were selected for relative quantification using the ΔCt method.

### Patient and control qPCR results

To further characterize the identified set of DDR genes we analysed their expression levels in AML patients and healthy controls and correlated them with the AML patients’ cytogenetic risk. The patients’ and controls’ characteristics are depicted in Table 1.

**Table 1:** Patient and control characteristics

(*N*)	AML (74)	Control (30)	*p*
Age, Years (Median (5–95% CI))	54 (26–68)	63.5 (23–84)	0.10
	AML		303
Karyotype (N)	Favorable (10)		304
	Intermediate (49)		305
	Unfavorable (11)		306
	Not Available (4)		307
Flt3-ITD Mut (N)	Not Detectable (41)		308
	Detectable (20)		309
	Not Available (13)		310
Npm1 Mut (N)	Not Detectable (37)		311
	Detectable (25)		312
	Not Available (12)		313
Response To Induction Chemotherapy	Complete Response (62)		314
	No Complete Response (12)		315
			316

**Fig. 2:**
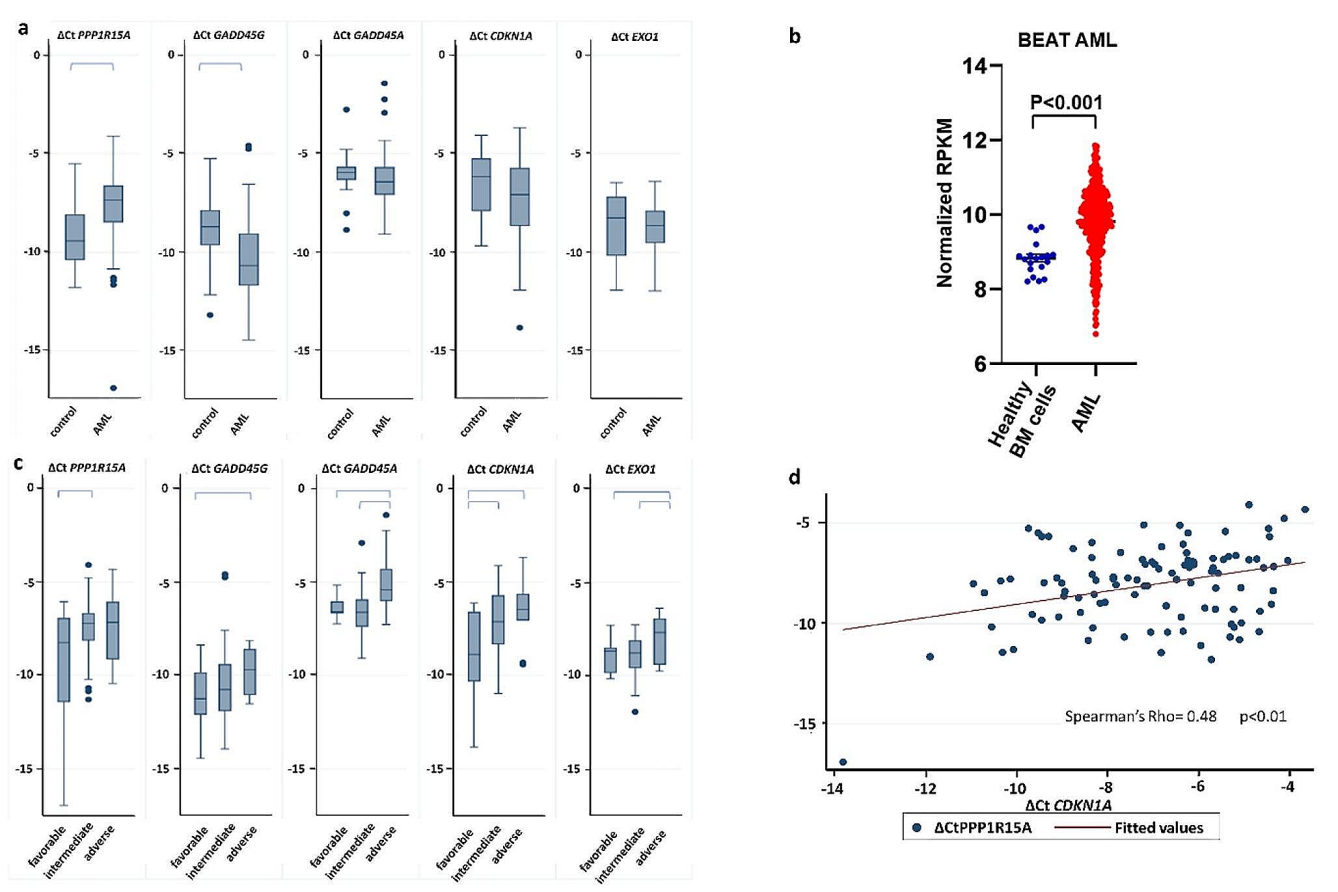
(**a**) Differential expression of DDR genes in AML patients and controls. Brackets denote *p* < 0.05 (**b**) *PPP1R15A* expression in healthy BM mononuclear cell samples and AML samples based on data from the BEAT AML dataset. (**c**) Differential expression of DDR genes in different cytogenetic AML risk groups. Brackets denote *p* < 0.05 (**d**) Correlation between *PPP1R15A* and *CDKN1A* in AML patients

The analysis of the expression level of the DDR genes demonstrated a marked increase in the expression of *PPP1R15A* as well as a decrease in the expression of *GADD45G* in AML patients compared to controls (*p* < 0.01 respectively). *GADD45A* and *CDKN1A* were also decreased in AML patients compared to controls, although not significantly (*p* = 0.076 and *p* = 0.088 respectively). *EXO1* expression was not different between AML patients and controls (Fig. 2a). To confirm our findings in an independent publicly available database we compared the *PPP1R15A* RNA levels between AML samples and healthy bone marrow mononuclear cell samples using data from the BEAT AML database [12]. We found that *PPP1R15A* is upregulated in AML samples compared to healthy bone marrow mononuclear cell samples (Fig. 2b). The correlation of expression of various genes showed a significant positive correlation between *PPP1R15A* and *CDKN1A* in AML patients (Fig. 2d). The analysis of DDR gene expression based on cytogenetic risk showed that all genes are upregulated in AML patients with higher cytogenetic risk. More specifically, *PPP1R15A* was upregulated in intermediate risk compared to favorable risk karyotype, *CDKN1A* was upregulated in intermediate and adverse karyotype compared to favorable risk karyotype, *GADD45A*, and *EXO1* were upregulated in adverse karyotype compared to favorable and intermediate risk karyotype and *GADD45G* was upregulated in adverse risk compared to favorable risk karyotype (Fig. 2c).

Our analysis showed no difference in the DDR gene expression levels between AML patients who responded to induction treatment and those who did not. No difference was found in the relative gene expression according to *FLT3-ITD* or *NPM1* mutational status either. There was finally no significant effect of gene expression on patients’ overall survival and event-free survival.

Overall, our analysis of human samples supports that PPP1R15A is the only gene out of the DDR genes upregulated in AML cells following Idarubicin and Cytarabine treatment that is also upregulated in AML patients and is associated with worse cytogenetic risk.

### IC50 values after treatment with idarubicin or cytarabine on AML cell lines

Our analysis based on the MTT assay showed that Idarubicin has an IC50 of 10 nM in the MOLM-13 cell line and 1 nM KASUMI-1 cells. Cytarabine, on the other hand, has an IC50 of 100 nM in both MOLM-13 and KASUMI-1 cells. All IC50 concentrations were recorded at 72 h post-treatment (Supplemental Figure S2). These results suggest that Idarubicin possesses a slightly stronger effect on cell viability than Cytarabine.

### Differential expression of PPP1R15A in MOLM-13 and KASUMI-1 cells after treatment with Idarubicin and cytarabine

**Fig. 3:**
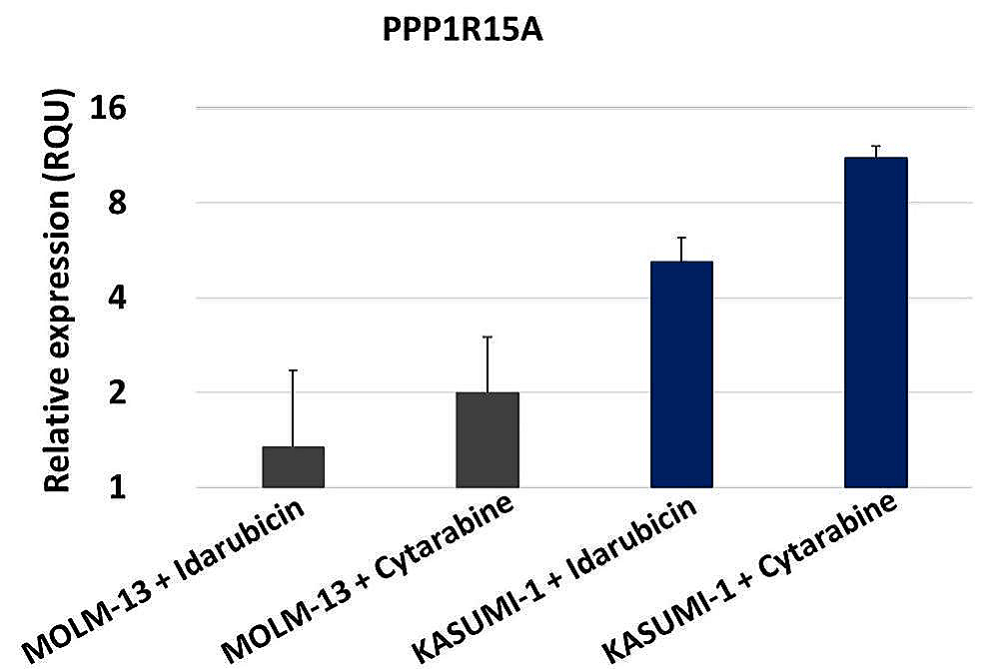
Differential Expression of *PPP1R15A* in MOLM-13 and KASUMI-1 cells after treatment with Idarubicin and Cytarabine

After 72 h of treatment, we observed a significant increase in the relative expression of *PPP1R15A* in both MOLM-13 and KASUMI-1 cells compared to untreated cells. Interestingly, the extent of *PPP1R15A* upregulation differed between the two cell lines. In MOLM-13 cells, treatment with the IC50 concentration of Idarubicin resulted in a moderate increase in *PPP1R15A* expression compared to untreated cells. Likewise, Cytarabine treatment induced a similar upward increase of *PPP1R15A* levels over the control. In the case of KASUMI-1 cells, both Idarubicin and Cytarabine treatments led to a substantial upregulation of *PPP1R15A* expression. Specifically, Idarubicin treatment resulted in a 5-fold increase in *PPP1R15A* levels compared to untreated cells. In contrast, Cytarabine treatment exhibited an even more remarkable effect, with *PPP1R15A* expression showing a robust 12-fold increase over the control. These findings indicate that both Idarubicin and Cytarabine modulate PPP1R15A expression in AML cells, with the KASUMI-1 cell line being more sensitive to the effect of the two chemotherapeutics (Fig. 3).

### Functional impact of PPP1R15A silencing on AML cell viability and response to Idarubicin or Cytarabine

To elucidate the functional significance of *PPP1R15A* in AML cell viability and chemotherapy response, we conducted *PPP1R15A* silencing experiments using siRNA in MOLM-13 and KASUMI-1 cell lines. Initially, we assessed the efficiency of *PPP1R15A* silencing by qPCR and western blot comparing the expression levels to cells transfected with scramble sequence.

**Fig. 4:**
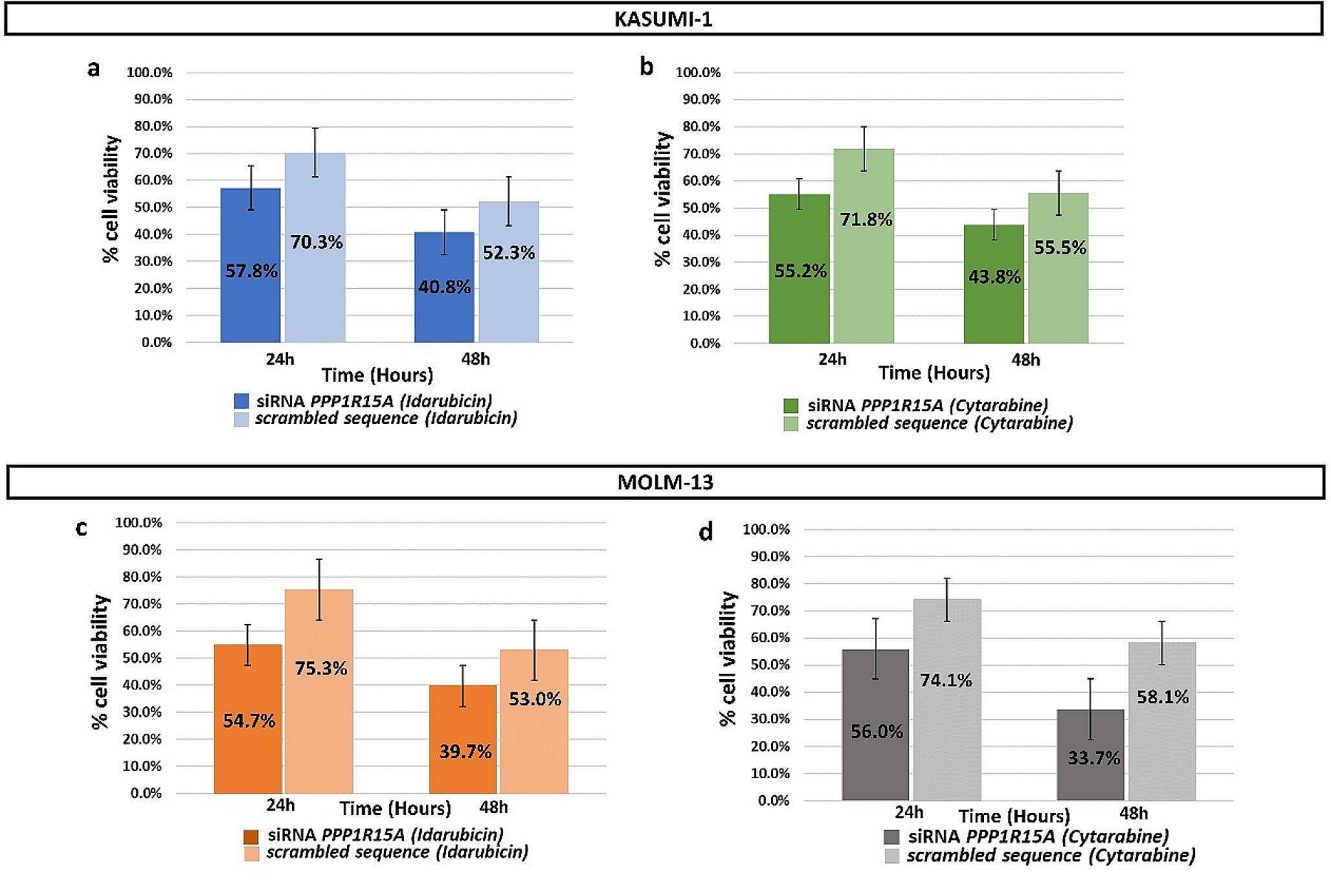
Cell viability after 24 h and 48 h of *PPP1R15A* silenced and scrambled cells. **a**: Kasumi-1, Idarubicin treated cell line, **b**: Kasumi-1, Cytarabine treated cell line, **c**: MOLM-13, Idarubicin treated cell line **d**: MOLM-13, Cytarabine treated cell line

In both MOLM-13 and KASUMI-1 cells, siRNA-mediated silencing resulted in a significant reduction in *PPP1R15A* expression levels compared to the scramble sequence (Supplemental Figure S3). After treatment of *PPP1R15A* silenced cells with Idarubicin or Cytarabine at their respective IC50 concentrations, we observed a marked decrease in cell viability in *PPP1R15A*-silenced cells following drug treatment compared to untreated cells. This reduction was evident in both MOLM-13 and KASUMI-1 cells (Fig. 4).

It is worth mentioning that cell viability did not significantly change in cell lines that had the PPP1R15A silenced compared to untransfected cells without treatment with Idarubicin or Cytarabine (Supplemental Figure S4).

### PPP1R15A knockdown by CRISPR-Cas9 increases the sensitivity of TF-1 cells to Idarubicin and Cytarabine

To further evaluate the implication of *PPP1R15A* expression in the sensitivity of AML cells to chemotherapy, CRIRPS-Cas9 mediated deletion of *PPP1R15A* was performed in the chemo-resistant TF-1 cells using two independent sgRNAs. Following puromycin selection, a single-cell clone was performed, and growing clones were screened by using qPCR for *PPP1R15A* (Supplemental Figure S5a). The *PPP1R15A* suppression at the protein level was confirmed by flow cytometry and western blot assay (Supplemental Figure S5b, c ).

TF-1 cells counting with trypan blue staining revealed no effect of *PPP1R15A* knockdown in cell growth (Supplemental Figure S6a) and methylcellulose-based clonogenicity assay showed no effect of *PPP1R15A* knockdown on cells colony formation (Supplemental Figure S6b).

**Fig. 5:**
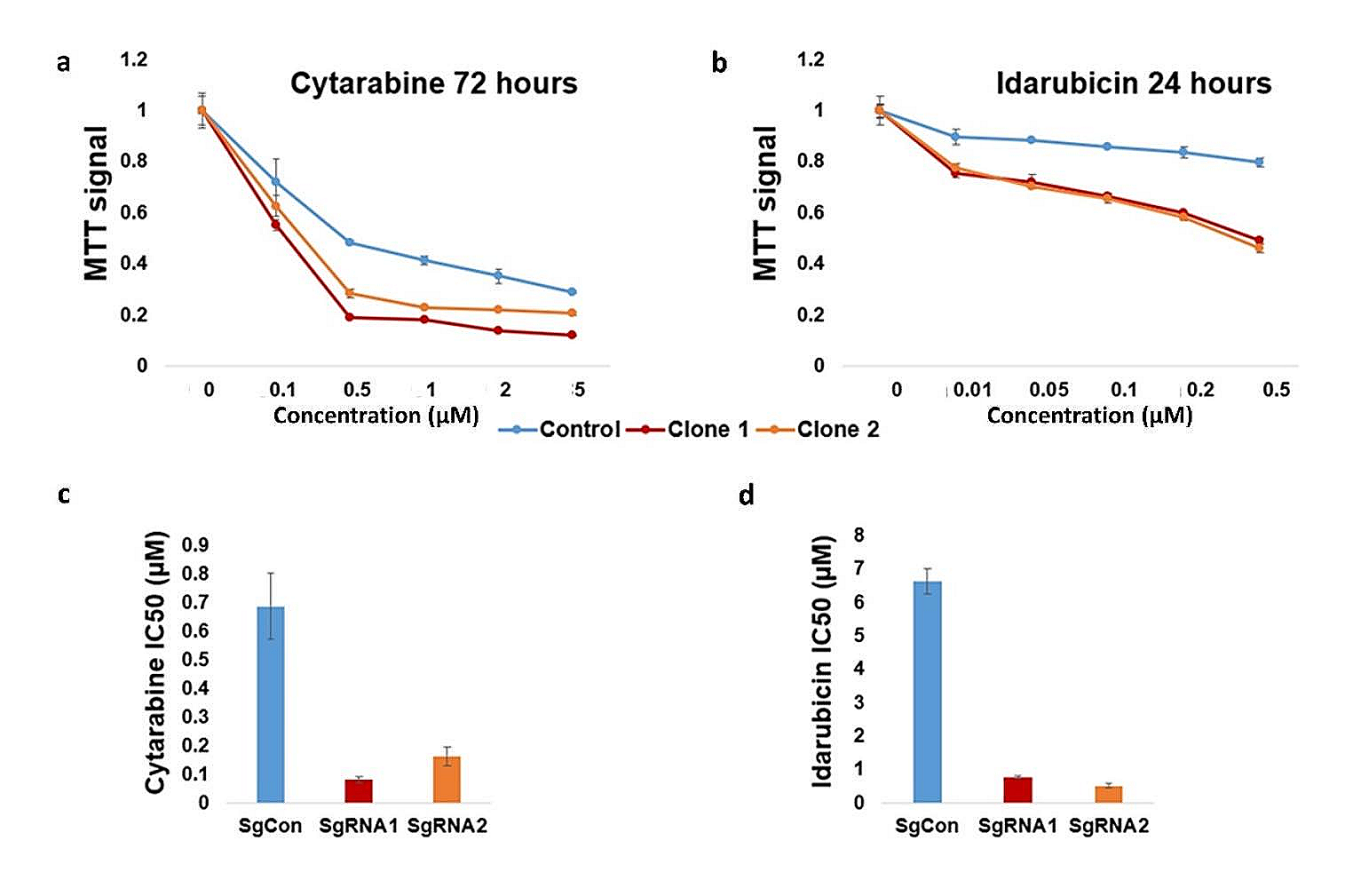
(**a**) TF-1 cells with PPP1R15A CRISPR-Cas9-mediated knockdown (clones 1 and 2) have a higher sensitivity to Cytarabine compared to control cells.(**b**) TF-1 cells with PPP1R15A CRISPR-Cas9-mediated knockdown (clones 1 and 2) have a higher sensitivity to Idarubicin compared to control cells. (**c**) TF-1 cells with PPP1R15A CRISPR-Cas9-mediated knockdown (clones 1 and 2) have lower IC50 dose for Cytarabine compared to control cells. (**d**) TF-1 cells with PPP1R15A CRISPR-Cas9-mediated knockdown (clones 1 and 2) have lower IC50 doses for Idarubicin compared to control cells

Control, clone 1 (sgRNA1), and clone 2 (sgRNA2) TF-1 cells were treated with 0–5 µM Cytarabine for 72 h and 0-0.5 Idarubicin µM for 24 h. *PPP1R15A* knockdown clones (clones 1 and 2) were significantly more sensitive to the chemotherapy agents compared to the control clone (Fig. 5a, b). Consistently, clones 1 and 2 had significantly lower IC50 for both Cytarabine and Idarubicin (Fig. 5c-d).

## Discussion

In this study, we examined the effect of Idarubicin and Cytarabine in the expression of a panel of DDR genes in AML cell lines. Genes that exhibited significant changes in their expression were chosen to be studied in bone marrow samples of AML patients and controls. Finally, we studied the effect of *PPP1R15A* downregulation, as this was the only DDR gene upregulated in AML patients compared to healthy controls and overexpressed in those with higher cytogenetic risk. We showed for the first time that its downregulation significantly increases AML cells’ chemosensitivity.

Using PCR arrays we observed an upregulation of of several DDR genes (*CDKN1A, GADD45A, GADD45G, EXO1*, and *PPP1R15A*) in KASUMI-1 and MV4-11 cell lines that survived following treatment with Idarubicin and Cytarabine. In malignancies genotoxic stress can be caused both by excessive DNA replication itself as well as by chemotherapy used to treat them [5]. This is in agreement with a positive association of these five DDR genes’ expression with increasing cytogenetic risk in AML patients in our study given that more complex karyotype has been linked with increased DNA damage. Furthermore, a previous study by Cavelier et al. has demonstrated an association of complex karyotype with increased DNA damage sensing through Histone 2X phosphorylation that resulted in checkpoint inhibition [13]. Overall, these findings suggest a link between genomic instability and upregulated DDR mechanisms. Of note, a higher level of genomic complexity can be detected in AML when genomic and transcriptomic data are used [14]. The association of genomic instability at high resolution with DDR would be very interesting to investigate.

*GADD45A*, *GADD45G*, and *GADD34* (also known as *PPP1R15A)* are part of the growth arrest and DNA damage–induced family of genes and they are involved in cell cycle arrest, DNA repair, cell survival, senescence, and apoptosis in response to oncogenic stress [15]. In our study, we found significant downregulation of *GADD45G* in AML patients compared to controls, as previously reported in a study by Guo et al. [16]. In keeping with their findings, we observed a tendency for shorter event-free survival in our patients who exhibited low *GADD45G* levels when we used a cut-off value of 45% *GADD45G* expression (*p* = 0.084, data not shown). While all 5 genes were overexpressed in samples from AML patients with intermediate and high cytogenetic risk compared to low cytogenetic risk, only *PPP1R15A* was found to be overexpressed in samples from AML patients compared to healthy controls highlighting its importance as a DDR mediator in this disease.

Our study reports for the first time the upregulation of *PPP1R15A* in AML patients compared to controls. Our results are further confirmed by data from the BEAT AML dataset. We also report enhanced chemosensitivity of AML cell lines after *PPP1R15A* downregulation. *PPP1R15A* is a holophosphatase comprised of two subunits, namely PPP1 and R15A. *PPP1R15A* is induced after treatment with DNA-damaging agents [17] and it enhances p53 independent apoptosis after treatment with some, but not all DNA damaging agents [18]. Interestingly, it was shown that in 11q23 AML, GADD34-mediated apoptosis is abrogated owing to the inactivating effect of the fusion protein on GADD34 [19]. These reports suggest that *PPP1R15A-induced* apoptosis is dependent both on the underlying disease as well as the DNA-damaging agent used.

In our study, it was demonstrated that *PPP1R15A* was overexpressed in AML probably as a result of genotoxic stress. In addition, its downregulation was associated with reduced cell viability in chemotherapy-treated AML cell lines indicating that *PPP1R15A* may have multiple mechanisms of action in DDR besides induction of apoptosis. This hypothesis is further supported by experimental data in solid tumors associating tumor hypoxia and autophagy with *PPP1R15A* upregulation, while *PPP1R15A* downregulation resulted in tumor growth suppression [20–23]. The mechanisms underlying *PPP1R15A* upregulation in AML remain to be elucidated.

The main function of *PPP1R15A* is the inactivation of the subunit of eukaryotic initiation factor 2 (eIF2α). *PPP1R15A* can bind to catalytic subunit protein phosphatase 1 (PP1c) and promote the dephosphorylation/inactivation of eIF2α. EIF2a is mainly activated under endoplasmic reticulum (ER) stress and it acts by inhibiting protein translation to restore proteostasis. Increased levels of eIF2α induce upregulation of *PPP1R15A* in order to restore translation and promote cell recovery [24–26]. *PPP1R15A* is currently being investigated for the treatment of protein-misfolding neurodegenerative diseases [27]. Furthermore, we found a significant correlation between *PPP1R15A* and *CDKN1A* expression in our patient cohort. *CDKN1A* is upregulated upon p53 activation after DNA damage, resulting in cell cycle arrest at G1 [28] and it has been reported that ER stress downregulates p21^CDKN1A^ levels, suggesting a link between ER stress and DDR [29].

*PPP1R15A* silencing studies, performed with two independent methods support that *PPP1R15A* suppression enhances the sensitivity of AML cells to Idarubicin or Cytarabine treatment, leading to reduced cell viability. Interestingly, untreated cells that were silenced for *PPP1R15A* did not show a decrease in cell viability suggesting that *PPP1R15A* silencing alone does not substantially affect cell survival under normal conditions. A previous study has shown that mice homozygous for a functionally inactivating mutation of *PP1R15A* exhibited normal phenotype [30]. These findings support that PPP1R15A inhibition could be an attractive therapeutic approach to increase the sensitivity of AML to induction chemotherapy.

## Conclusions

Overall, this study confirms the findings of previous investigators regarding DDR gene dysregulation in AML, while reporting for the first time the upregulation of *PPP1R15A* in this disease. Lastly, it highlights the functional relevance of *PPP1R15A* in AML cell viability and chemotherapy response.

By understanding the role of *PPP1R15A* in mediating drug sensitivity, we can potentially develop novel strategies to enhance the efficacy of chemotherapy and improve treatment outcomes for AML patients. Further studies are warranted to elucidate the molecular mechanisms underlying the interaction between *PPP1R15A* and chemotherapy agents, paving the way for the development of targeted therapies in AML.

### Electronic supplementary material

Below is the link to the electronic supplementary material.

Supplementary Material 1

## Data Availability

Data will be made available upon request to the corresponding author.
