# Supplementary material for: Silencing of the DNA damage repair regulator PPP1R15A sensitizes acute myeloid leukemia cells to chemotherapy
Source: Ann Hematol. 2024 Jun 6;103(8):2853–63. doi: 10.1007/s00277-024-05785-x (PMC11283411; doi:10.1007/s00277-024-05785-x)
Supplement: Supplementary file 1 — Supplementary Material 1 [file 277_2024_5785_MOESM1_ESM.docx]

**Supplemental Files**

**Silencing of the DNA damage repair regulator *PPP1R15A* sensitizes acute myeloid leukemia cells to chemotherapy**

Anthi Bouchla^1^, Christina D. Sotiropoulou^2^, Christopher Esteb^3^, Theodoros Loupis^4^, Sotirios G. Papageorgiou^1^, Gina Deliconstantinos^1^, Maria Pagoni^5^, Eleftheria Hatzimichael^6^, Maria Dellatola^5^, Smaragdi Kalomoiri^5^, Elisavet Apostolidou^6^, Christos K. Kontos^2^, Theodoros Karantanos^*^, Vasiliki Pappa^1^ (T.K. and V.P. contributed equally to this study)

**Methods**

**Table S1.** The sequences used for transfection assay.

| **oligo** | **Primer sequence (5ʹ🡪 3ʹ)** |
| --- | --- |
| siRNA | AAGGCTGTGTACGCTGTCACG |
| Negative control (scrambled) | GTGCGCATGCCTTCGGTAAGA |

**Table S2.** The QuantiTect Primers

| **Gene** | **QuantiTech** |
| --- | --- |
| *PPP1R15A* | QT00013321 |
| *CDKN1A* | QT00062090 |
| *EXO1* | QT00080717 |
| *GADD45G* | QT00200557 |
| *GADD45A* | QT00014084 |
| *GAPDH* | QT00079247 |

**Table S3.** The primer pairs were used to amplify *PPP1R15A*.

| **Gene** | **Direction** | **Primer sequence (5ʹ🡪 3ʹ)** |
| --- | --- | --- |
| *PPR115A* | Sense | GATTCAGAAGCAGCCTTGGG |
|  | Antisense | ATAGATGGCCACTCGGAAGG |
| *GAPDH* | Sense | ATGGGGAAGGTGAAGGTCG |
|  | Antisense | GATATTGTTGCCATCAATGACCC |
| *HPRT1* | Sense | TGGAAAGGGTGTTTATTCCTCAT |
|  | Antisense | TTGCTGACCTGCTGGATTACAT |
| *B2M* | Sense | ACTGAATTCACCCCCACTGA |
|  | Antisense | TCCAAATTCTGCTTGCTTGCTT |

**Results**

**
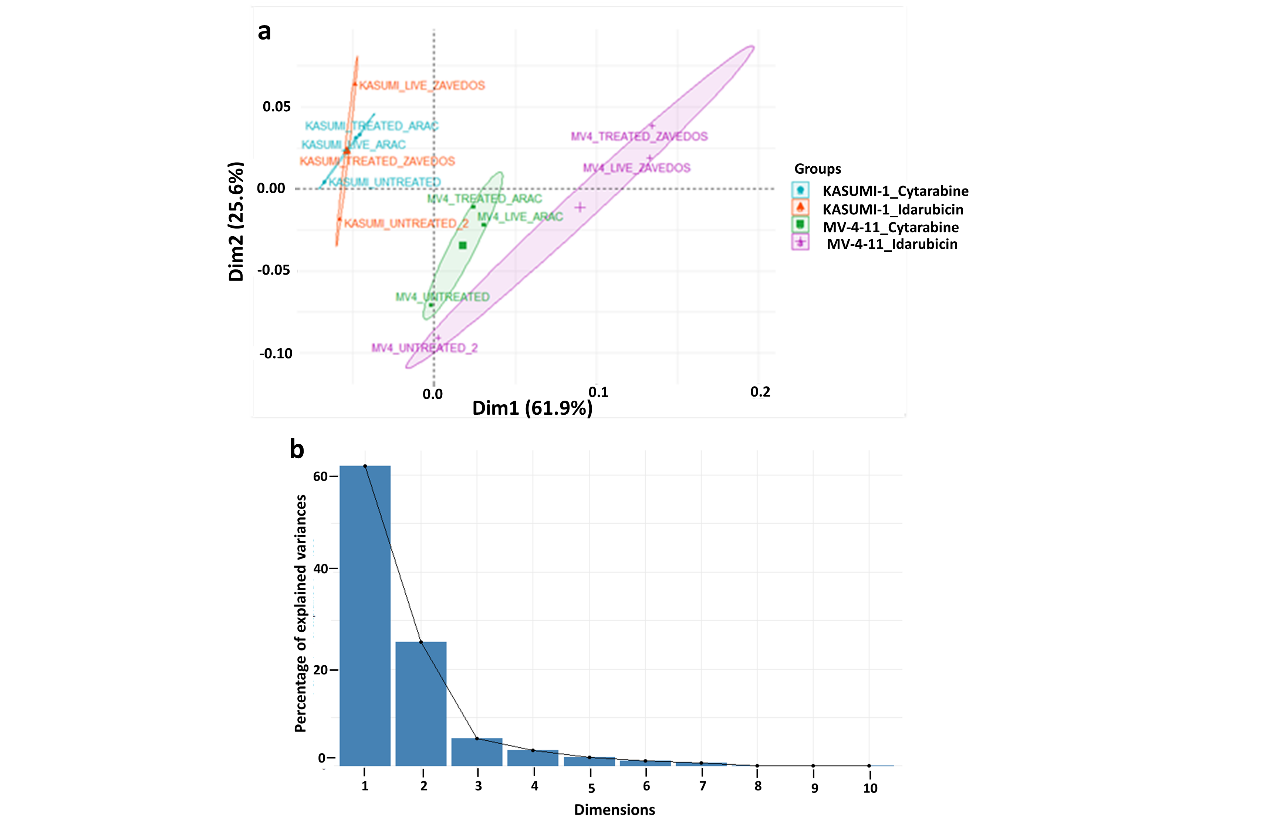
**

**Figure S1. PCA analysis of differential DDR gene expression in chemotherapy treated AML cell lines** (a) PCA plot and (b) scree plot of DDR gene expression in MV4-11 and KASUMI-1 cell lines before and after treatment with Idarubicin and Cytarabine.

**
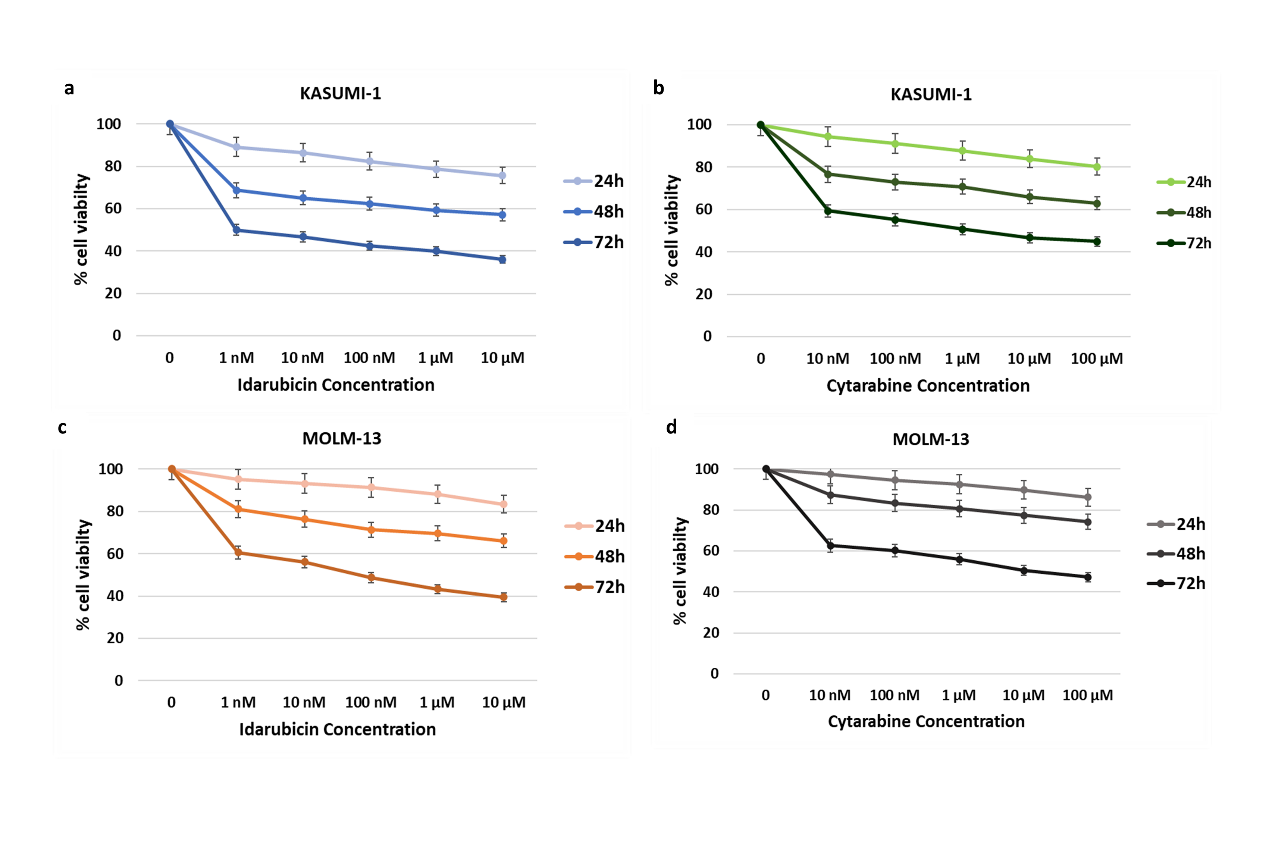
**

**Figure S2. IC50 determination in chemotherapy-treated AML cell lines.** Scatter plots of the MTT assay results of % viability *vs* time for each drug concentration in MOLM-13 and KASUMI-1 cell lines.

**
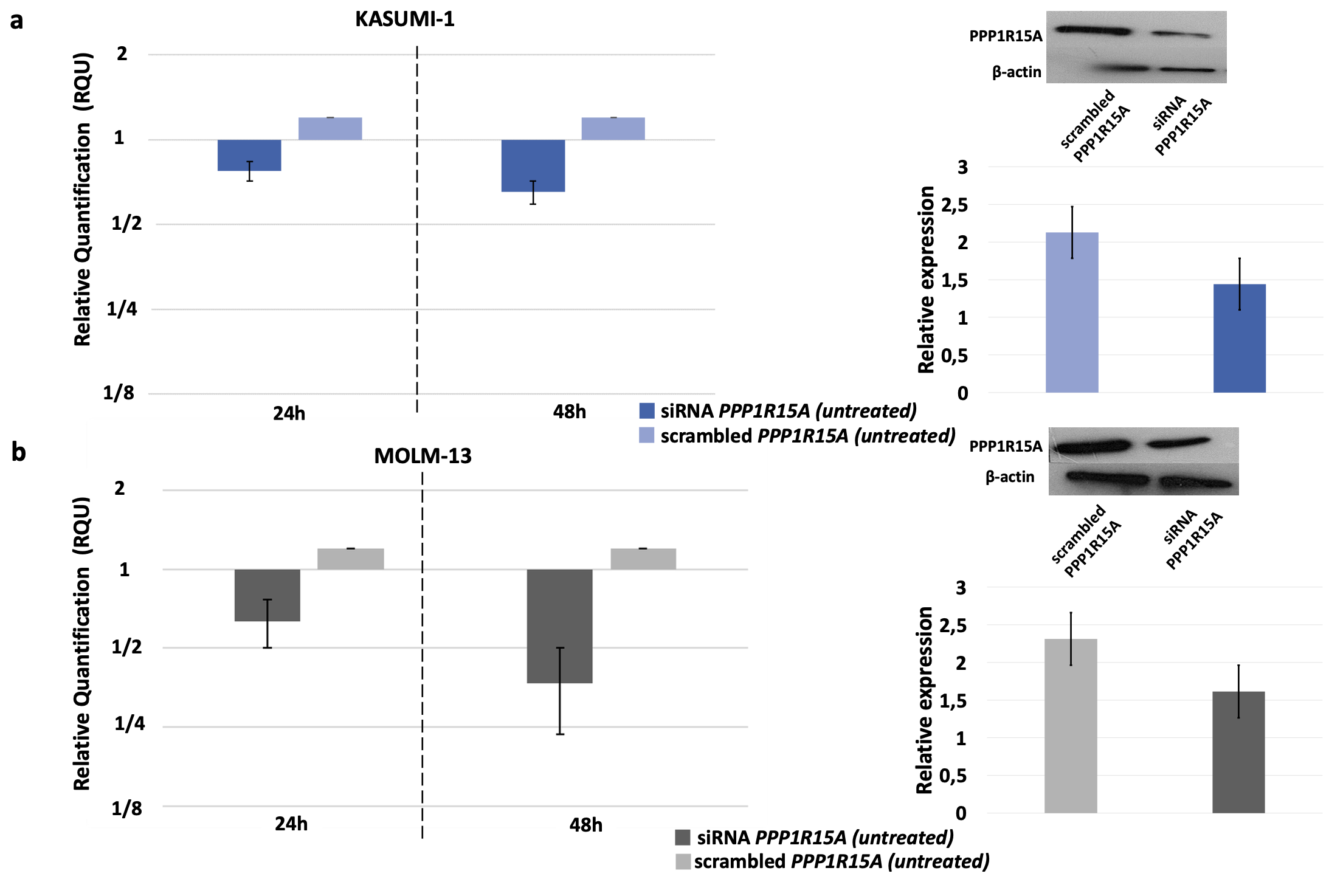
**

**Figure S3.** **Effective *PPP1R15A* silencing in AML cell lines**. The transfection of KASUM1-1 and MOLM-13 with siRNA resulted in the effective silencing of the *PPP1R15A* gene.


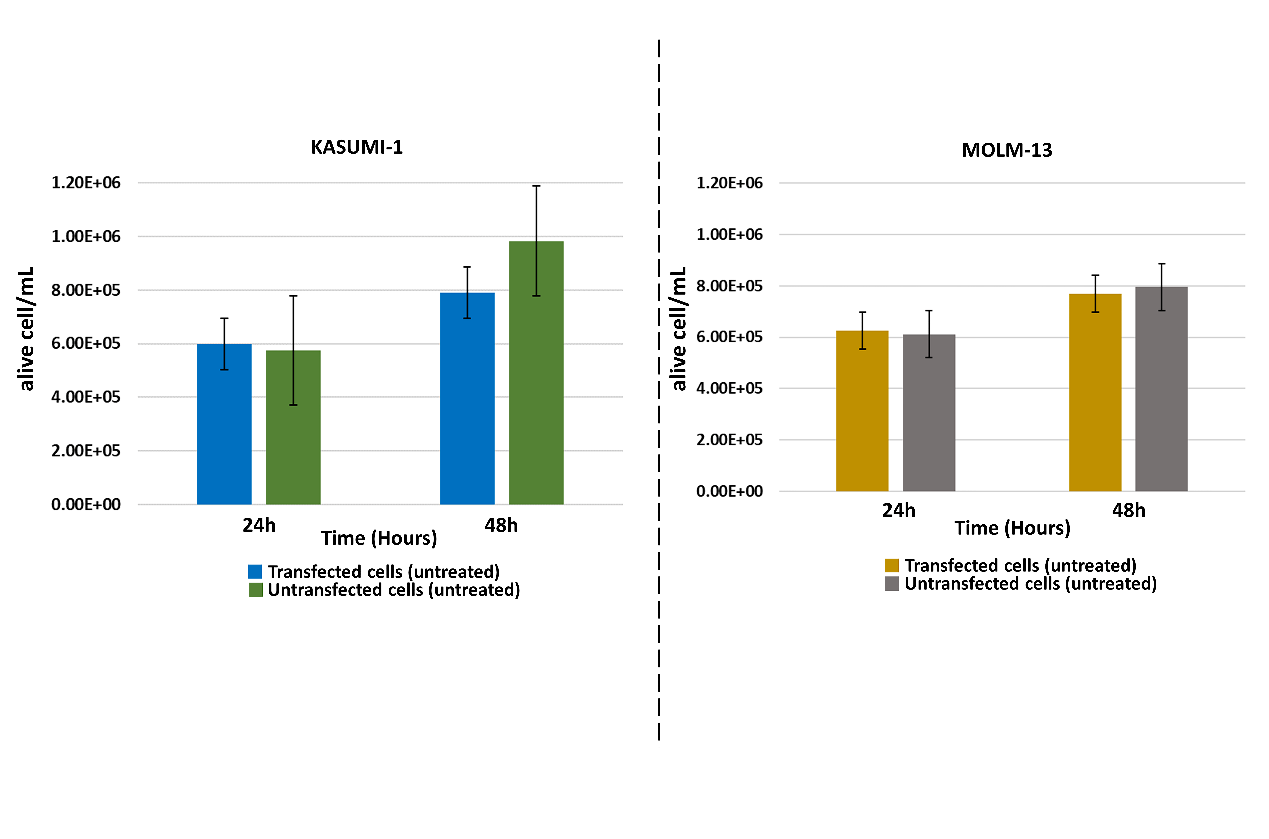


**Figure S4. *PPP1R15A* silencing in AML cell lines did not affect cell viability in the absence of chemotherapy.** The number of alive cells at 24h and 48h after silencing of *PPP1R15A* is similar to the number of alive cells expressing *PPP1R15A* at 24h and 48h, without treatment in KASUM1-1 and MOLM-13 cell lines.

**
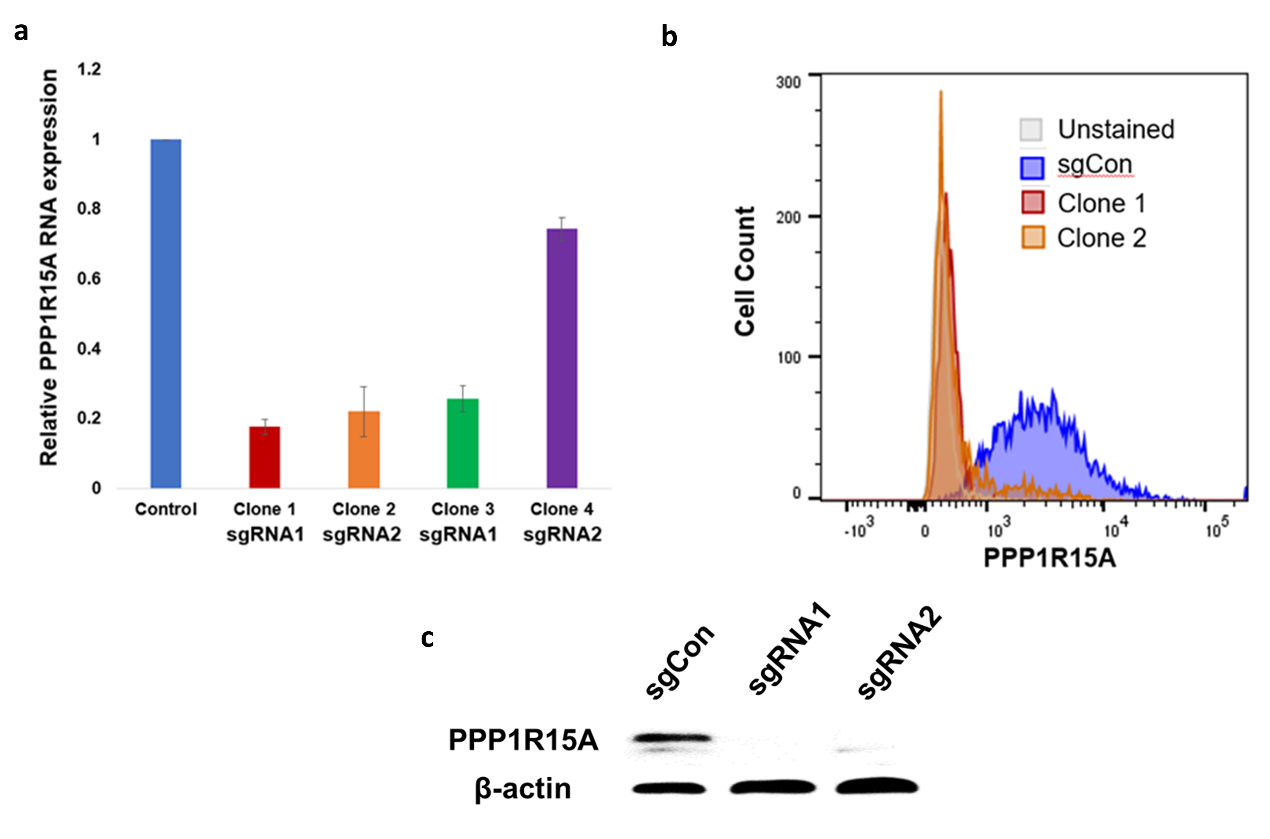
**

**Figure S5. Effective *PPP1R15A* knockdown in TF-1 clones** (a) qPCR used to identify TF-1 clones with *PPP1R15A* knockdown. (b, c**)** Flow cytometry and Western Blot were used to confirm the *PPP1R15A* knockdown in TF-1 clones at the protein level.**
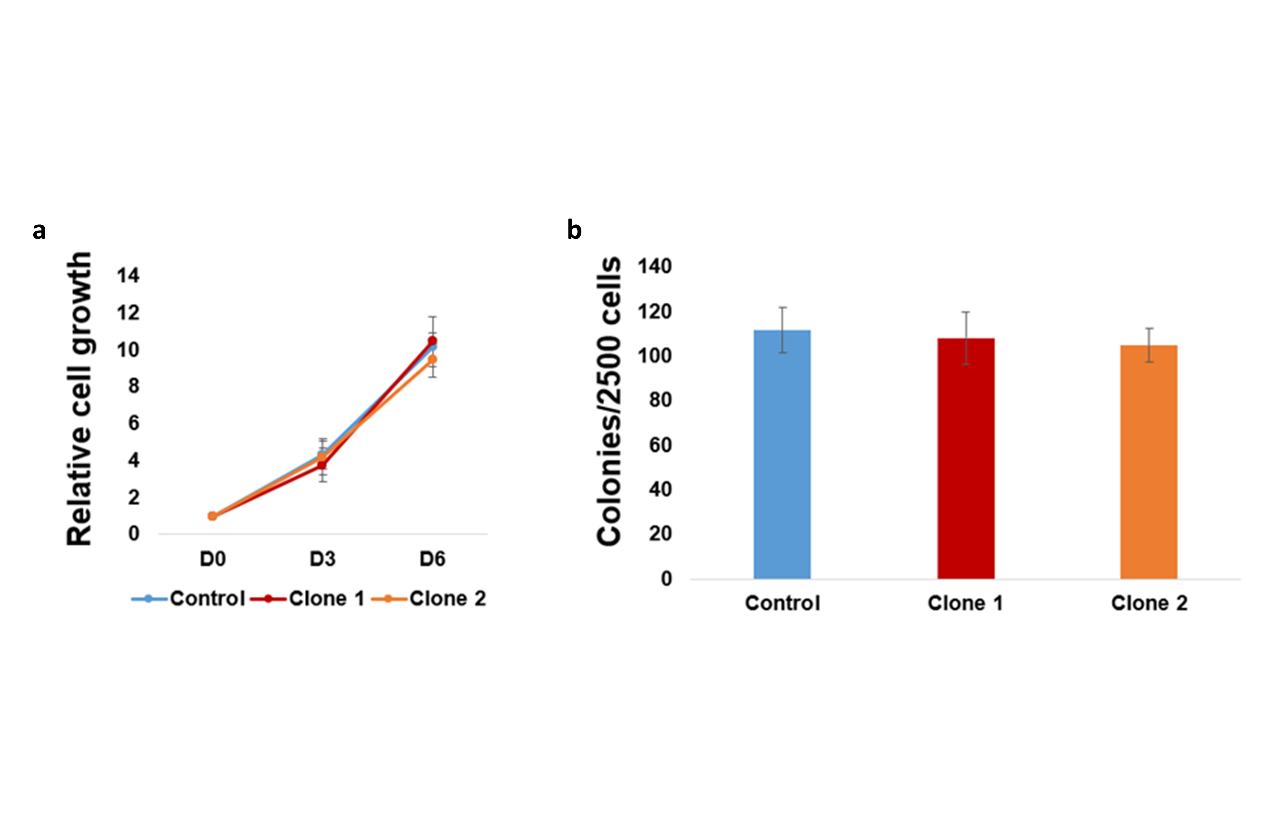
**

**Figure S6. *PPP1R15A* knockdown in an AML cell line did not affect cell viability in the absence of chemotherapy.**  (a) Cell counting with trypan blue showed no effect of *PPP1R15A* knockdown in cells’ growth. (b**)** Methylcellulose base clonogenicity assay showed no effect of *PPP1R15A* knockdown in cells’ colony formation.
